# CMG2 Expression Is an Independent Prognostic Factor for Soft Tissue Sarcoma Patients

**DOI:** 10.3390/ijms18122648

**Published:** 2017-12-07

**Authors:** Thomas Greither, Alice Wedler, Swetlana Rot, Jacqueline Keßler, Astrid Kehlen, Hans-Jürgen Holzhausen, Matthias Bache, Peter Würl, Helge Taubert, Matthias Kappler

**Affiliations:** 1 Center for Reproductive Medicine and Andrology, Martin Luther University, 06120 Halle (Saale), Germany; alice.wedler@student.uni-halle.de; 2 Department of Oral and Maxillofacial Plastic Surgery, Martin Luther University Halle-Wittenberg, 06120 Halle (Saale), Germany; swetlana.rot@medizin.uni-halle.de (S.R.); matthias.kappler@medizin.uni-halle.de (M.K.); 3 Department of Radiotherapy, Martin Luther University Halle-Wittenberg, 06120 Halle (Saale), Germany; jacqueline.kessler@medizin.uni-halle.de (J.K.); matthias.bache@medizin.uni-halle.de (M.B.); 4 Institute of Medical Microbiology, Martin Luther University Halle-Wittenberg, 06120 Halle (Saale), Germany; astrid.kehlen@medizin.uni-halle.de; 5 Institute of Pathology, Martin Luther University Halle-Wittenberg, 06120 Halle (Saale), Germany; hans-juergen.holzhausen@medizin.uni-halle.de; 6 Department of General and Visceral Surgery, Hospital Dessau, 06847 Dessau-Roßlau, Germany; peter.wuerl@klinikum-dessau.de; 7 Clinic of Urology, FA University Hospital Erlangen-Nuremberg, 91054 Erlangen, Germany; Helge.Taubert@uk-erlangen.de

**Keywords:** CMG2, soft tissue sarcoma, pathobiology, outcome

## Abstract

The capillary morphogenesis gene 2 (CMG2), also known as the anthrax toxin receptor 2 (ANTXR2), is a transmembrane protein putatively involved in extracellular matrix (ECM) adhesion and tissue remodeling. CMG2 promotes endothelial cell proliferation and exhibits angiogenic properties. Its downregulation is associated with a worsened survival of breast carcinoma patients. Aim of this study was to analyze the CMG2 mRNA and protein expression in soft tissue sarcoma and their association with patient outcome. CMG2 mRNA was measured in 121 tumor samples of soft tissue sarcoma patients using quantitative real-time PCR. CMG2 protein was evaluated in 52 tumor samples by ELISA. CMG2 mRNA was significantly correlated with the corresponding CMG2 protein expression (r_s_ = 0.31; *p* = 0.027). CMG2 mRNA expression was associated with the mRNA expressions of several ECM and tissue remodeling enzymes, among them CD26 and components of the uPA system. Low CMG2 mRNA expression was correlated with a worsened patients’ disease-specific survival in Kaplan-Meier analyses (mean patient survival was 25 vs. 96 months; *p* = 0.013), especially in high-stage tumors. A decreased CMG2 expression is a negative prognostic factor for soft tissue sarcoma patients. CMG2 may be an interesting candidate gene for the further exploration of soft tissue sarcoma genesis and progression.

## 1. Introduction

Soft tissue sarcomas are a heterogeneous group of tumors probably arising from a transformed mesenchymal progenitor cell [1,2]. Actually, soft tissue sarcomas are defined by the adult tissue they resemble according to the WHO (World Health Organization) 2013 Classification of Sarcomas of the Soft Tissue and Bone [3]. The incidence is low, with around 6:100,000 persons between 1973 and 2006 [4]; however, the survival rate is still low with around 50% after 5 years and 30% after 15 years [4]. In particular, metastasization is still an urging clinical issue [5]. Soft tissue sarcomas metastasize preferentially into the lung and the brain, with around 20% of patients having developed metastases at diagnosis [6]. As the genetic and phenotypical heterogeneity of soft tissue sarcoma currently hinder a proper prognostic evaluation, independent molecular prognostic markers are greatly needed.

CMG2 is a transmembrane protein initially identified as being upregulated during endothelial cell tube morphogenesis in three-dimensional (3D) matrices [7]. It is the second known receptor for the anthrax toxin, leading to the synonymous abbreviation ANTXR2 [8]. Mutations in CMG2 are linked to hyaline fibromatosis syndrome characterized, besides others, by the accumulation of hyaline material in connective tissues [9,10]. This syndrome is characterized by a loss of CMG2 function resulting from the mutation-mediated retention of CMG2 in the endoplasmic reticulum [11,12]. Furthermore, besides binding laminin and collagen IV with high affinity [7], CMG2 was recently identified as a key regulator of collagen VI turnover [13]. This points towards an essential role of CMG2 in ECM (extracellular matrix) adhesion and remodeling, both biological processes linked to metastasis. Concordantly, a low CMG2 mRNA expression was shown to be an independent negative prognostic marker in breast carcinoma patients [14], and a knockdown of CMG2 significantly increased the invasiveness of prostate carcinoma cell lines [15]. However, further prognostic data are rare.

One striking example for ECM remodeling pathways being metastasis-related is the uPA (urokinase-type plasminogen activator) system. It consists of the cell surface-bound uPA receptor (uPAR), the secreted ligand uPA, and the inhibitor PAI-1 (plasminogen activator inhibitor 1). An increased expression of the uPA system components induces cell migration and invasion and is associated with the epithelial/mesenchymal transition characterizing the metastatic process [16,17]. In this context, overexpression of the uPA system is correlated to a worsened prognosis in a variety of tumors, including but not exclusively colorectal cancer [18], pancreatic cancer [19], breast cancer [20], and soft-tissue sarcomas [21].

Thus, the aim of this study was to analyze the prognostic impact of intratumoral CMG2 mRNA and protein levels in soft tissue sarcoma patients. Additionally, we tested the association of CMG2 mRNA expression with the expression levels of several metastasis- and angiogenesis-related genes previously studied by us in the actual patient cohort [22,23,24].

## 2. Results

### 2.1. CMG2 mRNA and Protein Expression in Soft Tissue Sarcoma

**Figure 1:**
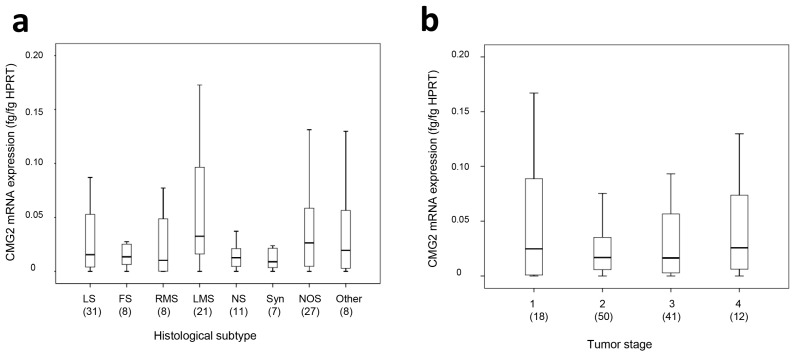
Box-Plot overview on CMG2 mRNA expression in the different soft tissue sarcoma histological subtypes (**a**) and tumor stages (**b**). Numbers in bracket depict the cases (*n*) per category. Median CMG2 mRNA expression was slightly, but not significantly higher in leimosarcoma and NOS (**a**), and in stage 1 or stage 4 tumors (**b**). Abbreviations: LS—liposarcoma; FS—fibrosarcoma; RMS—rhabdomyosarcoma; LMS—leiomyosarcoma; NS—neuronal sarcoma; Syn—synovial sarcoma; NOS—not other specified.

CMG2 mRNA was detected in the patient samples with a mean concentration of 0.1 fg/fg HPRT (hypoxanthine-guanine phosphoribosyltransferase) (range: 0.0–3.96 fg/fg HPRT). CMG2 protein expression was measured in 52 samples, with a mean concentration of 0.79 ng/µg total protein (range: 0.09–1.47 ng/µg total protein). In a separate analysis of the different histological subtypes, the median expressions of the CMG2 mRNA in liposarcoma, fibrosarcoma, rhabdomyosarcoma, synovial sarcoma, and neuronal sarcoma were relatively similar (0.01–0.015 fg/fg HPRT), while the median CMG2 mRNA expression in leiomyosarcoma and NOS (not other specified) was slightly, but not significantly, increased (0.032 fg/fg HPRT and 0.026 fg/fg HPRT, respectively, see Figure 1a). Considering the different tumor stages, the median CMG2 mRNA expression in stage 2 and 3 tumors (0.017 and 0.016 fg/fg HPRT, respectively) was slightly, but also not significantly, lower than in stage 1 or stage 4 tumors (0.025 and 0.026 fg/fg HPRT, respectively, see Figure 1b).

For the demographic and clinical parameters of the patient cohort, see Table 1. For survival analyses, CMG2 mRNA expression and CMG2 protein expression were separated into two or four groups, according to the median or the quartiles (median: CMG2 mRNA: 0.018 fg/fg HPRT; CMG2 protein: 0.74 ng/µg total protein; 25% quartiles: CMG2 mRNA: 0.004 fg/fg HPRT; CMG2 protein: 0.57 ng/µg total protein; 75% quartiles: CMG2 mRNA: 0.05; CMG2 protein: 1.05 ng/µg protein) as the cut-off values. In initial analyses, we found that especially the quartile of patients with the lowest CMG2 mRNA expression exhibited a remarkably negative prognosis, therefore for subsequent survival analyses, a cut-off value of 0.004 fg CMG2/fg HPRT was chosen.

### 2.2. Association of CMG2 mRNA to Angiogenesis- and Metastasis-Related Genes

**Figure 2:**
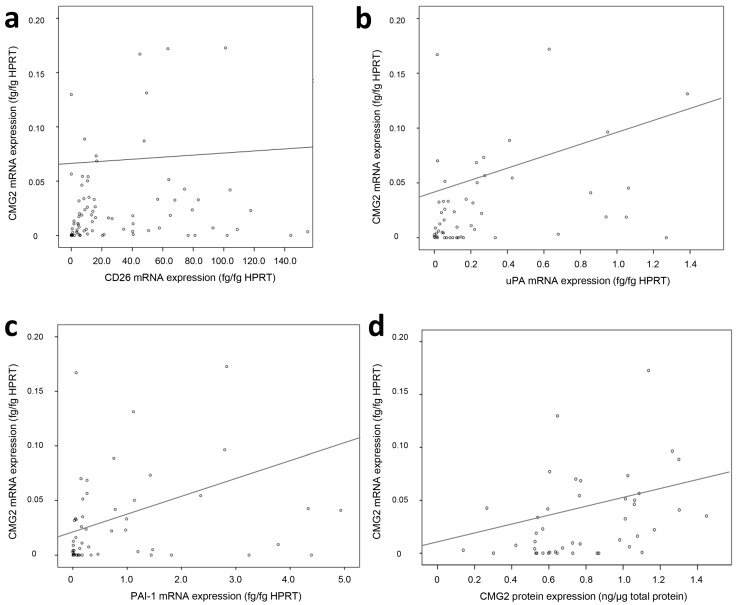
Bivariate correlation analyses of CMG2 mRNA expression and CD26 mRNA (**a**), uPA mRNA (**b**), PAI-1 mRNA (**c**) and CMG2 protein (**d**) expression.

In bivariate regression analyses according to Spearman-Rho (see Table 2), CMG2 mRNA expression was significantly associated to the mRNA expression of the dipeptidyl peptidase 4 family member CD26 (r_s_ = 0.26; *p* = 0.005, see Figure 2a).

Furthermore, CMG2 mRNA expression was significantly associated with those of different components of the uPA/PAI system (uPA: r_s_ = 0.37; *p* = 0.001; PAI-1: r_s_ = 0.36; *p* = 0.002; see Figure 2b,c; uPAR: r_s_ = 0.27; *p* = 0.023). Additionally, CMG2 mRNA expression was inversely correlated to LGR5 mRNA expression (r_s_ = −0.21; *p* = 0.041) and miR-199a-5p expression (r_s_ = −0.29; *p* = 0.008). CMG2 mRNA expression was also significantly associated with CMG2 protein expression in soft tissue sarcoma samples (r_s_ = 0.31; *p* = 0.027, see Figure 2d).

### 2.3. CMG2 Expression and Patient Survival

**Figure 3:**
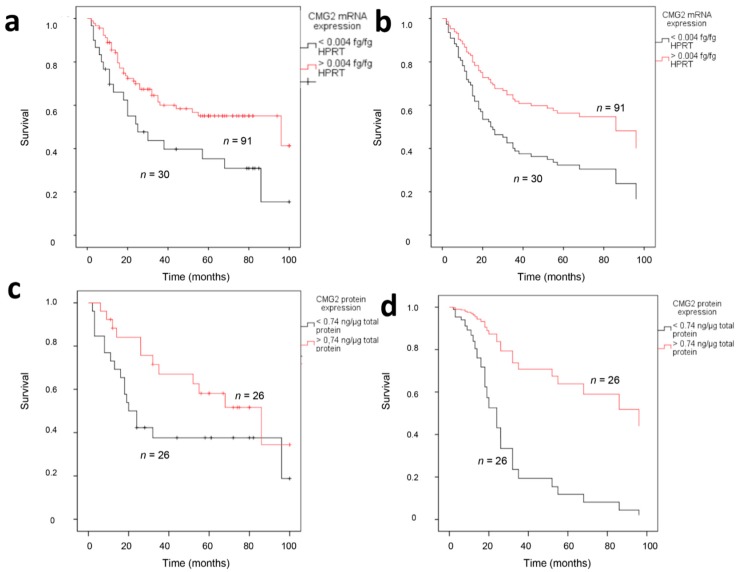
Survival analyses for CMG2 mRNA expression and protein expression in soft tissue sarcoma patients. Kaplan-Meier survival analyses revealed a significant worsened prognosis for patients with a low CMG2 mRNA expression in their tumors (**a**), but not for protein expression (**c**). Multivariate Cox’s regression analyses exhibited the detrimental effect of low CMG2 mRNA (**b**) and CMG2 protein (**d**) expression on patients’ disease-specific survival.

In Kaplan-Meier survival analyses, CMG2 mRNA expression was significantly associated with patients’ disease-specific survival, with a median survival time of 25 months for soft tissue sarcoma patients with a low CMG2 mRNA expression (*n* = 30), in comparison to 90 months for patients with an elevated CMG2 mRNA expression (*n* = 91; *p* = 0.013; log-rank test; see Figure 3a). A multivariate Cox’s regression analysis adjusted to tumor localization, tumor stage, tumor entity, and resection mode revealed a 2.12-fold increased risk of cancer-related death for patients with a low CMG2 mRNA expression (*p* = 0.019; see Figure 3b). CMG2 protein expression was not significantly associated with soft tissue sarcoma patient survival (*p* = 0.07; log-rank test; see Figure 3c); however, the patient group with a low CMG2 protein expression also exhibited a decreased median survival time of 20 months in comparison to 86 months for patients with a high CMG2 protein expression. Furthermore, in multivariate Cox’s regression analyses, patients with a low CMG2 protein expression exhibited a significant 4.7-fold increased risk for tumor-related death (*p* = 0.047; see Figure 3d). Additionally, sub-analyses were employed on the prognostic impact of the CMG2 mRNA expression in patients with tumors of different histological subtypes or different stages. While CMG2 mRNA exhibited no significant association with the patients’ outcome in the different histological subclasses, patients with high-stage tumors (3 + 4) and lower CMG2 mRNA expression exhibited a significant worsened survival than high-stage soft tissue sarcoma patients with elevated CMG2 mRNA expression (11 versus 23 months median survival time in Kaplan-Maier survival analyses).

### 2.4. CMG2 mRNA Expression and Survival in Other Tumor Entities

**Figure 4:**
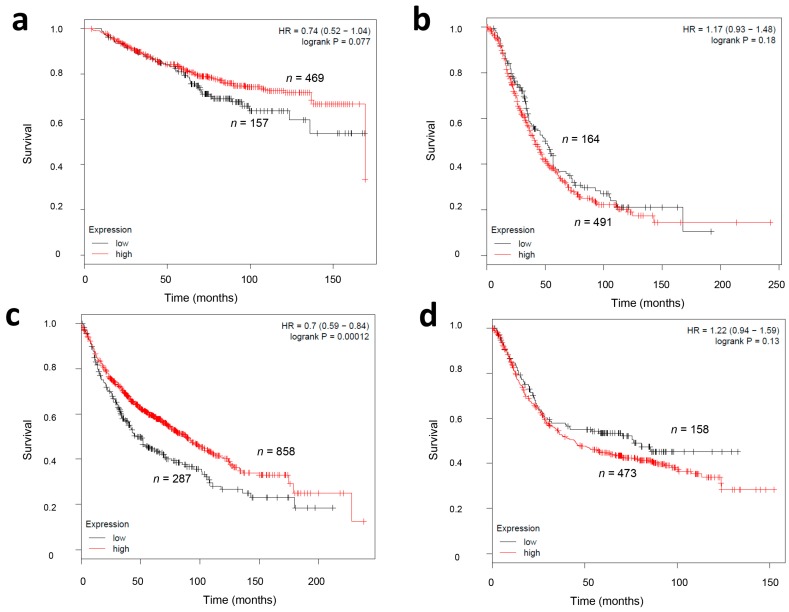
Comparative data on to the impact of CMG2 mRNA expression on the overall survival of breast (**a**), ovarian (**b**), lung (**c**), and gastric (**d**) cancer patients. Plots were drawn by the Kaplan-Meier Plotter algorithm (Available online: http://kmplot.com/analysis) facilitating microarray data of the CMG2/ANTXR2 probe 225524_at. Abbreviations: HR—hazard ratio.

Next, we analyzed the association of CMG2 transcript levels and overall survival in other tumor entities as breast, lung, gastric, and ovarian cancer by studying CMG2 mRNA microarray data in an online survival analyses tool (Kaplan-Meier Plotter, available online: http://kmplot.com/analysis/) [21]. A low CMG2 mRNA expression (defined as the lowest quartile of the analyzed samples) was significantly associated with a worsened survival in lung carcinoma patients (*p* = 0.0001; log-rank test; see Figure 4c). Univariate Cox’s regression analyses in these tumor entities showed a 1.4-fold increased relative risk for patients with a low CMG2 mRNA expression. In breast carcinoma patients, a low CMG2 mRNA expression was also correlated with a worsened overall survival rate (relative risk RR = 1.35, see Figure 4a); however, this result showed only a trend towards significance. On the other hand, in ovarian or gastric cancer patients, no significant association between a low CMG2 mRNA expression and patients’ survival was detectable (*p* = 0.13 and *p* = 0.18, respectively; log-rank-test, see Figure 4b,d).

## 3. Discussion

In this study, the mRNA and protein expression of CMG2 in soft tissue sarcoma were analyzed regarding their impact on patient survival. We could demonstrate that CMG2 mRNA is detectable in the majority of the tumor specimens, and that its decreased expression is associated with worsened disease-specific survival of soft tissue sarcoma patients.

Initially, we detected a significant association of the CMG2 mRNA expression with the mRNA expression of the dipeptidyl-peptidase 4 family member CD26 and with the mRNA expressions of components of the uPAR-PAI system. CD26 is a serine integral membrane protease which is involved in tissue remodeling and putatively in metastasis. Recently, the induction of CD26 has been demonstrated to be involved in the re-organization of human adipose tissue [25]. Additionally, the expression of CD26 has been identified immunohistochemically in a wide range of sarcomas, independent of their malignant potential [26]. Concordantly, the expression of components of the uPA system in soft tissue sarcomas was proven immunohistochemically [27] and at the mRNA level [22]. Furthermore, as it was shown in the actual soft tissue sarcoma patient cohort, the elevated expression of combinations of uPA, uPAR, or PAI protein in the tumor tissue as well as in the serum of patients was significantly associated with a worsened survival [28].

Furthermore, CMG2 mRNA expression was inversely correlated to LGR5 mRNA expression, a G-protein coupled receptor mainly engaged in Wnt signaling [29]. LGR5 was found to be overexpressed in several solid malignant tumors, having a close association with initiation and recurrence of different cancer types and correlating with tumor growth, invasion, and poor outcome [30,31,32,33]. In the actual soft tissue sarcoma patient cohort, we previously demonstrated that a low mRNA level of a LGR5 splice variant lacking exon 5 (LGR5Δ5) was correlated to a poor prognosis for the disease-associated survival and to a shorter recurrence-free survival, while LGR5 full-length mRNA expression exhibited no correlation to patient prognosis [23].

Interestingly, CMG2 mRNA expression was inversely correlated to miR-199a-5p expression, which points towards a post-transcriptional regulation of the CMG2 transcript by this microRNA. However, an in silico analysis (Available online: www.targetscan.org) exhibited no direct regulation of CMG2 by miR-199a-5p or -3p or its cluster mate miR-214. Therefore, an indirect regulation by the miR-199a-mediated downregulation of a transcription factor or inducer of CMG2 is more likely than a direct regulation of the CMG2 transcript. For instance, miR-199a targets the transcription factor Ets-1 in breast carcinoma and hence downregulates the expression of β 1-integrin, leading to an increase in the invasive abilities of the tumor cell [34]. Furthermore, miR-199a was demonstrated to downregulate Smad4 expression and subsequently negatively regulate the TGF-β signaling pathway [35]. However, the tumor-biological role of miR-199a is still under debate and seems to be highly dependent on the tumor entity. While miR-199a overexpression is detected in progressed gastric cancer [36] and highly invasive melanoma [37], in renal cancer [38] or testicular cancer miR-199a is considered a tumor suppressor gene [39,40]. In soft tissue sarcoma, we observed a significant association between low miR-199a-5p expression and a worsened patient survival [24]. This seemingly contradictory result underlines the need for further research on the regulation of CMG2 mRNA transcription and the interaction between CMG2 and proliferative and invasive signaling pathways.

It is necessary to state that a limit of most of the previous correlations is the low correlation coefficient (<±0.3 in case of CD26, uPAR, miR-199a, and LGR5). This may be due to expression variations of different genes in ex vivo samples, for instance in consequence of allelic loss or altered epigenetic/post-transcriptional regulation. Nonetheless, there is evidence on potential interaction effects between some of the described molecular factors, as for instance CD26 and uPA were demonstrated to cluster together in specialized membrane domains and act synergistically on the migration of endothelial cells during the angiogenic process [41]. However, the mechanisms and pathways putatively underlying the described correlation between CMG2 and CD26, uPA/uPAR/PAI, miR-199a, or LGR5 remain to be elucidated in further research.

The main problem in assessing the impact of CMG2 in sarcoma genesis and progression lays in the still lacking data on the physiological function of this gene and the signaling pathways involved, beside the fact that CMG2 has been linked to several cellular processes. For instance, in endothelial cells, the downregulation of CMG2 results in a significant reduction of murine endothelial cell proliferation, while migration is not affected [42]. In breast cancer cell lines, a CMG2 knockdown results in a decreased proliferation and increased adherence of the cells [14]. In contrary, a decreased adherence and no effects on proliferation due to a CMG2 knockdown was observed in prostate carcinoma cells [15]. However, there is growing evidence of a predominant role of CMG2 in extracellular matrix homeostasis. CMG2 mutations revealed that CMG2 protein is essential in mouse parturition by regulating the uterine collagen homeostasis [43], a function facilitated by CMG2 for instance by the mediation of collagen VI uptake [13]. However, the way in which the matrix remodeling function of CMG2 is essential for angiogenesis, invasion, or tumor formation and progression has not yet been comprehensively studied.

The correlation of CMG2 expression with tumor patient survival is not well studied so far. In the actual study, we demonstrated a significant association between low CMG2 mRNA and protein expression and a worsened survival of soft tissue sarcoma patients, underlined by a similar effect of low CMG2 mRNA expression in lung and breast cancer patients. Ye and colleagues showed that CMG2 transcripts were reduced in breast tumor samples, and that CMG2 mRNA expression tended to be inversely associated with an increased tumor stage [14]. Furthermore, low CMG2 mRNA expression was significantly associated with decreased disease-free and overall survival in breast cancer patients [14]. Additionally, CMG2 transcripts at relatively high levels were detected in prostate cancer tissues in comparison to normal prostatic tissue; however, no statement on the association of an altered mRNA expression on patient survival was made in this study [15]. On the other hand, a CMG2-knockdown resulted in a decreased matrix adhesion in prostate carcinoma cells [15]. Interestingly, although the exact role of CMG2 in tumorigenesis and progression is not well described, the interest in CMG2’s angiogenic properties as targets for anti-tumor therapy are high, resulting in several reports on inhibitors putatively useful for treatment [44,45,46,47,48]. Furthermore, we demonstrated that a lower CMG2 mRNA expression was significantly associated with a worsened survival, especially in high-stage (3 + 4) soft tissue sarcomas, while there was no significant association between CMG2 mRNA expression and survival in low-stage tumors. One may speculate that a lowered CMG2 expression may not be detrimental in well-differentiated tumors, but may support metastasis synergistically in a cellular environment, where many pro-metastatic mutations and genetic alterations have accumulated.

In conclusion, low CMG2 mRNA expression is significantly associated with a worsened prognosis in soft tissue sarcoma patients. The associations of CMG2 mRNA expression to the mRNA expressions of CD26 as well as components of the uPA system warrant further research on the role and regulation of CMG2 in soft tissue sarcoma genesis and progression.

## 4. Materials and Methods

### 4.1. Patients

One hundred and twenty-one soft tissue sarcoma patients were included in this study, which underwent surgical resection of their tumor between 1998–2001 at the Department of Surgery, University of Leipzig (Leipzig, Germany) without prior adjuvant treatment. Patients and tissue specimen cryopreservation have been described in prior studies [49,50]. The study was approved by the local ethics committee of the Medical Faculty of the Martin Luther University Halle-Wittenberg and the Medical Faculty of the University of Leipzig (Decision from the 24.01.2007). All patients gave written consent.

### 4.2. RNA Isolation

Total RNA was isolated from 5-µm tissue slices (5 µm each). Briefly, the tissue was incubated in Trizol (Thermo Fisher Scientific, Waltham, MA, USA) for 5 min at room temperature, then mixed with chloroform (AppliChem, Darmstadt, Germany) and centrifuged. The aqueous phase was collected, and subjected to a DNAse (Qiagen, Hilden, Germany) digestion. Subsequently, the total RNA was precipitated with isopropanol (AppliChem, Darmstadt, Germany) for 12 h at 4 °C and washed with ethanol solutions (96% and 70%). RNA was dissolved in RNAse-free water (Qiagen, Hilden, Germany). RNA concentrations were assessed spectrometrically.

### 4.3. cDNA Synthesis and qPCR

The cDNA synthesis was performed with a RevertAid First strand cDNA synthesis kit (Thermo Fisher Scientific, Waltham, MA, USA). Briefly, 1 µg RNA was used for cDNA synthesis with random hexamer primers in a Thermo-Trioblock TB1 (Biometra, Göttingen, Germany). cDNA was amplified with Quantitect (Qiagen, Hilden, Germany) in a quantitative real-time reaction for CMG2 and HPRT (reference gene) with the following primers: CMG2 fw: 5′-TCC TGC AGA AGA GCC TTT-3′; CMG2 rw: 5′-CTG CTA ATG ATG GCA CCA GA-3′; HPRT fw: 5′-TTG CTG ACC TGC TGG ATT AC-3′; HPRT rw: 5′-CTT GCG ACC TTG ACC ATC TT-3′. Samples were run on a RotorGene real-time-PCR cycler (LTF Labortechnik, Wasserburg, Germany). CMG2 mRNA expression was calculated as absolute values (fg/fg HPRT) according to a standard curve generated by the use of a dilution series of the gene primer-specific amplificates.

### 4.4. Protein Isolation and ELISA

Total protein was isolated by lysation of the tissue samples with RIPA buffer containing Tris-HCl (50 mM, pH 8.0; AppliChem, Darmstadt, Germany), 200 mM NaCl (Sigma-Aldrich, St. Louis, MI, USA), 1 mM ethylene diamine tetraacetic acid (EDTA; AppliChem, Darmstadt, Germany), 1 mM ethylene glycol tetraacetic acid (EGTA; AppliChem, Darmstadt, Germany), 0.25% deoxycholate (AppliChem, Darmstadt, Germany), and protease inhibitor cocktail (1:100; Sigma-Aldrich, St. Louis, MI, USA). Protein concentrations were measured by Bradford reagent (BioRad, Hercules, CA, USA), and 1 µg was used for the ELISA procedures. ELISA were performed using the ANTXR2 ELISA kit (antibodies-online, Aachen, Germany) according to the manufacturer’s protocol. In brief, 1 µg of total protein from 47 tissue specimens each were applied to specific wells and incubated for 1 h at 37 °C. Wells were washed and incubated with a primary antibody (1 h at 37 °C), washed again and incubated with a secondary antibody. CMG2 protein concentration was measured by substrate change-induced absorbance at 450 nm. All protein concentrations were normalized to the applied protein amount (µg total protein).

### 4.5. Statistics

Statistics were performed with SPSS 20.0 (IBM, Ehingen, Germany). Data were analyzed with bivariate correlation analyses according to Spearman-Rho and Chi Square tests. Survival analyses were performed with Kaplan-Meier analyses, as well as univariate and multivariate Cox’s regression analyses adjusted for the confounders resection status, localization of tumor, tumor size, and tumor stage. Additional Kaplan-Meier survival analyses for CMG2 mRNA expression in breast, lung, ovarian, and gastric carcinoma samples were carried out by the Kaplan-Meier Plotter algorithm (Available online: http://kmplot.com/analysis/) [51].

## Figures and Tables

**Table 1:** Clinical and histopathological characteristics in relation to the CMG2 mRNA expression.

Parameter	Description	CMG2 Low Expression (<0.004 fg/fg HPRT)	CMG2 High Expression (>0.004 fg/fg HPRT)	Chi^2^ Test (*p* Value)
Age	<60 years	19	45	n.s.
	>60 years	11	46	
Gender	female	11	41	n.s.
	male	19	50	
Patient status	alive	10	54	0.013
	deceased	20	37	
Tumor stage ^a^	I	6	12	n.s.
	II	8	42	
	III	13	28	
	IV	3	9	
Resection	radical (R0)	19	68	n.s.
	not radical (R1)	11	23	
Tumor localisation	extremities	19	60	n.s.
	trunk wall	3	8	
	head/neck	2	2	
	abdomen/retro-peritoneum	5	20	
	multiple locations	1	1	
Histological subtype	LS	8	23	n.s.
	FS	1	7	
	RMS	3	5	
	LMS	3	18	
	NS	3	8	
	Syn	2	5	
	NOS	6	21	
	Other	4	4	
Tumor size	T1	3	15	n.s.
	T2	27	76	
Number of relapses	0	20	56	n.s.
	1	4	19	
	>2	6	16	

^a^ Union for International Cancer Control Guidelines; Abbreviations: LS—Liposarcoma; FS—Fibrosarcoma; RMS—Rhabdomyosarcoma; LMS—Leiomyosarcoma; NS—Neuronal sarcoma; Syn—Synovial sarcoma; NOS—not other specified; T1—<5 cm in diameter; T2—>5 cm in diameter; n.s—not significant.

**Table 2:** Bivariate correlation analyses (Spearman-Rank). Significant correlations of the mRNA expression of CMG2 with those of other tumor-associated genes and with CMG2 protein levels in the analyzed soft tissue sarcoma cohort.

Parameter	r_s_	*p*	*n*
CD26 mRNA	0.26	0.005	117
uPA mRNA	0.37	0.001	73
PAI-1 mRNA	0.36	0.002	73
uPAR mRNA	0.27	0.023	73
LGR5 mRNA	−0.21	0.041	92
miR-199a	−0.29	0.008	85
CMG2 protein	0.31	0.027	52
